# An Antiviral Defense Role of AGO2 in Plants

**DOI:** 10.1371/journal.pone.0014639

**Published:** 2011-01-31

**Authors:** Jagger J. W. Harvey, Mathew G. Lewsey, Kanu Patel, Jack Westwood, Susanne Heimstädt, John P. Carr, David C. Baulcombe

**Affiliations:** Department of Plant Sciences, University of Cambridge, Cambridge, United Kingdom; Ecole Normale Superieure, France

## Abstract

**Background:**

Argonaute (AGO) proteins bind to small-interfering (si)RNAs and micro (mi)RNAs to target RNA silencing against viruses, transgenes and in regulation of mRNAs. Plants encode multiple AGO proteins but, in *Arabidopsis*, only AGO1 is known to have an antiviral role.

**Methodology/Principal Findings:**

To uncover the roles of specific AGOs in limiting virus accumulation we inoculated turnip crinkle virus (TCV) to *Arabidopsis* plants that were mutant for each of the ten *AGO* genes. The viral symptoms on most of the plants were the same as on wild type plants although the *ago2* mutants were markedly hyper-susceptible to this virus. *ago2* plants were also hyper-susceptible to cucumber mosaic virus (CMV), confirming that the antiviral role of AGO2 is not specific to a single virus. For both viruses, this phenotype was associated with transient increase in virus accumulation. In wild type plants the AGO2 protein was induced by TCV and CMV infection.

**Conclusions/Significance:**

Based on these results we propose that there are multiple layers to RNA-mediated defense and counter-defense in the interactions between plants and their viruses. AGO1 represents a first layer. With some viruses, including TCV and CMV, this layer is overcome by viral suppressors of silencing that can target AGO1 and a second layer involving AGO2 limits virus accumulation. The second layer is activated when the first layer is suppressed because AGO2 is repressed by AGO1 via miR403. The activation of the second layer is therefore a direct consequence of the loss of the first layer of defense.

## Introduction

RNA silencing is a natural antiviral defense mechanism in plants in which Argonaute (AGO) proteins use bound small-interfering (si)RNAs to target cleavage or translational suppression of complementary RNA. In plants the siRNAs are generated by Dicer-like (DCL) proteins that cleave longer double stranded precursor RNAs. Plant viruses encode suppressor proteins of RNA silencing as counter-defense mechanisms that influence the accumulation and spread of viruses in infected plants [1]. There are also RNA silencing pathways that target transposons and endogenous mRNAs and, correspondingly, there are multiple DCL and AGO proteins encoded by different members of multigene families. One of the variant RNA silencing pathways that targets endogenous mRNA involves microRNAs that are similar to siRNAs but with a distinct biogenesis pathway [2].

In *Arabidopsis thaliana* the four plant DCL proteins generate virus-derived small interfering RNAs (vsiRNAs), with DCL1 being specific to DNA viruses [3], [4], [5], [6]. There are ten Argonaute (AGO) proteins and several of them have been implicated in antiviral RNA silencing by several lines of evidence: AGO1 [7], AGO2 and AGO5 [8] proteins bind vsiRNAs; *AGO1* is up-regulated upon virus infection [7]; *ago1* mutants are hyper-susceptible to cucumber mosaic virus (CMV); *AGO2* is induced by viral silencing suppressors [9], [10]; and *ago1* and *ago7* mutant plants are hyper-susceptible to silencing suppressor-minus mutant turnip crinkle virus (TCV) [11]. However, only one of these examples with *ago1* and CMV, provides evidence that an AGO protein protects against a fully virulent virus [12].

To further investigate the antiviral role of AGO proteins we monitored TCV-induced symptoms on a panel of *Arabidopsis* plants that are mutant for each of the ten AGO proteins and found that an *ago2-1* mutant was hyper-susceptible to TCV. Further investigation confirmed and characterized an antiviral defense role for AGO2 with both TCV and CMV but not with tobacco mosaic virus (TMV).

## Results

A panel of homozygous *Arabidopsis* plants mutant for each AGO protein was screened for hyper-susceptibility to TCV, a positive strand RNA virus in the genus *Carmovirus*. Its coat protein (CP) – P38 – is a silencing suppressor [13] and TCV lacking a functional P38 (TCVΔCP) is unable to spread systemically in *Arabidopsis* [14].

**Figure 1:**
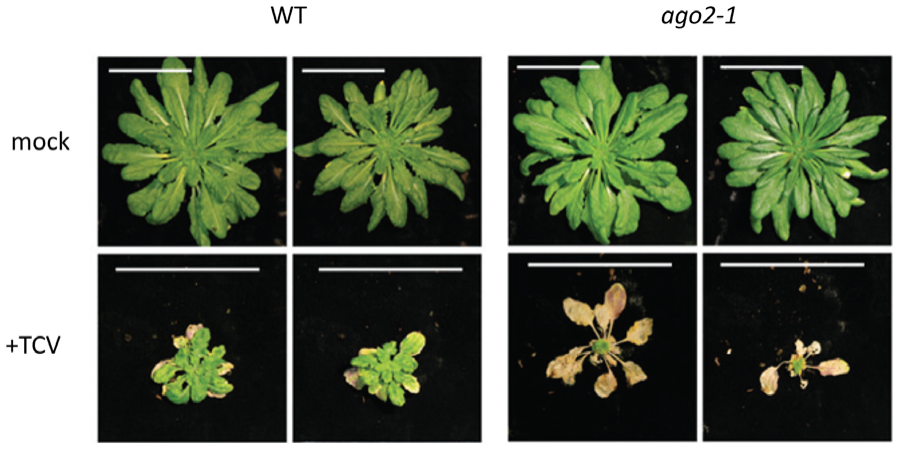
*ago2-1* is hyper-susceptible to TCV. At 49dpi (depicted above) the *ago2-1* plants exhibited far more severe symptoms than infected wild type (WT) controls and had ceased growing. These *ago2-1* plants eventually died (by 56dpi). Plants were inoculated at the 6–8 true leaf stage. Scale bars are 5 cm.

TCV symptoms in most of the homozygous mutant plants were no more severe than those in the wild type plants. The *ago1-25* plants were highly stunted and chlorotic but the non inoculated plants had a growth phenotype and the differential effect of the virus was probably no more than on the wild type plants. However, *ago2-1* plants grew normally when not infected but they exhibited more severe symptoms than wild type plants (Figure 1) when infected with TCV. These enhanced symptoms were observed consistently in all TCV-infected plants in five independent trials with at least five plants of each genotype per treatment in each.

At 3–20 days post inoculation (dpi) with TCV the symptoms in *ago2-1* mutants were enhanced chlorosis and anthocyanin accumulation relative to a wild type plant that spread from the inoculated to the systemically infected leaves. By 14–35 dpi there was necrosis in the mutant but not the wild type plants and eventually the mutant plants died (Figure 1).

**Figure 2:**
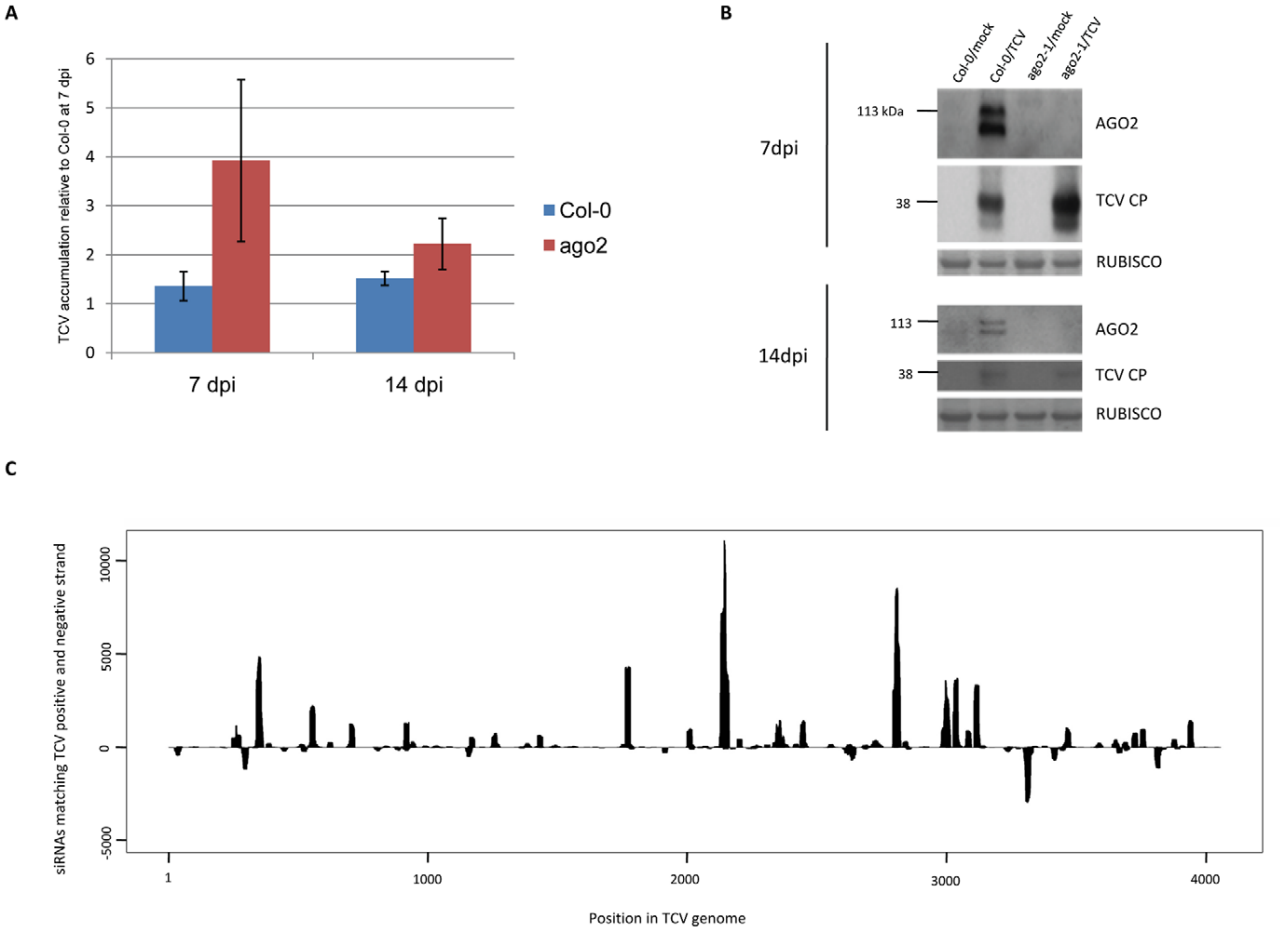
AGO2 is induced by TCV, affects accumulation of viral RNA and coat protein and binds viral siRNA. **a**) Q-RT-PCR of TCV accumulation in WT and ago2-1 plants. **b**) Western blots of AGO2 and TCV CP accumulation in mock- and TCV-infected WT (Col-0) and *ago2-1* plants, as indicated at 7 and 14dpi. Ponceau S-stained RUBISCO large subunit serves as a loading control. **c**) TCV siRNAs bound to AGO2, sequenced by Illumina, derived from along the TCV genome. Those above the x-axis match the TCV positive strand, while those below the x-axis match the negative strand.

To find out whether these enhanced symptoms correlated with levels of virus we used quantitative RT-PCR. This analysis revealed that the TCV RNA was more abundant in the *ago2-1* mutants than in the wild type controls after 7 dpi but by 14 dpi there was no difference between the two types of plant (Figure 2a) despite the very marked difference in symptoms. This pattern of a transient increase in the *ago2-1* mutant was also confirmed by western blotting (Figure 2b).

The AGO2 protein could not be detected by western blotting in wild type plants that had not been inoculated. However, after infection with TCV, the AGO2 antibody detected two proteins of 113kDa (the predicted size of AGO2) and 108 kDa (Figure 2b). These proteins were absent in the *ago2-1* mutant plants indicating that they represent isoforms of AGO2 due possibly to posttranslational modification or cleavage by proteolytic enzymes. The TCV-induced accumulation of AGO2 was observed consistently in six independent replicates in two experiments and it persisted until at least 14dpi (Figure 2b).

In principle the antiviral effect of AGO2 could be because this protein binds to endogenous siRNAs or miRNAs that target suppressors of defense. Alternatively it could be because AGO2 binds to viral siRNAs that target the viral RNAs directly. We favour the latter possibility because sequencing of siRNAs bound to AGO2 of TCV infected plants includes many TCV-specific siRNAs that are predominantly from the viral positive RNA strand (Figure 2c).

**Figure 3:**
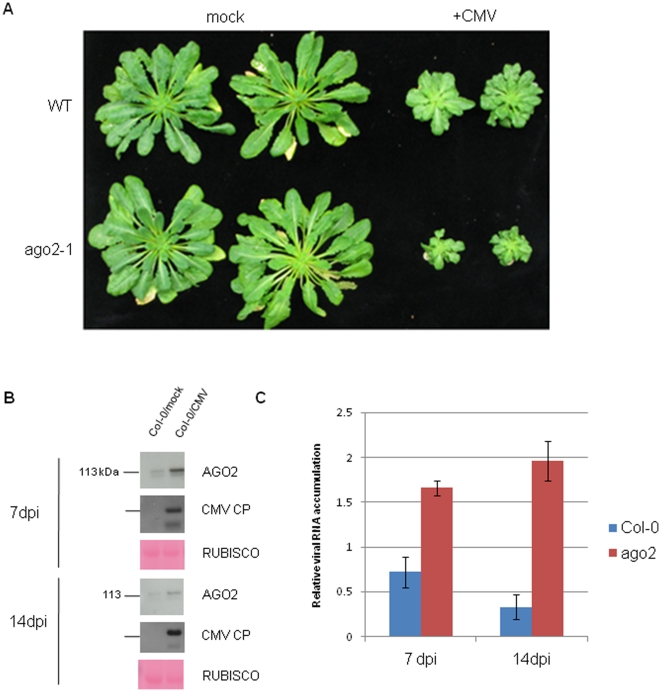
The antiviral role of AGO2 is not specific to TCV. **a**) Growth of CMV-infected *ago2-1* mutant plants is more stunted than in WT (Col-0) controls. Plants were inoculated at the 6–8 true leaf stage and photographed at 56dpi. **b**) Western blot of AGO2 and CMV CP accumulation in mock- and CMV-infected WT (Col-0) and *ago2-1* plants, as indicated at 7 and 14dpi. Ponceau S-stained RUBISCO large subunit serves as the loading control. **c**) Q-RT-PCR of CMV accumulation.

The *ago2-1* and wild type plants were equally susceptible to tobacco mosaic virus (TMV; genus *Tobamovirus*). However the stunting and mosaic symptoms of CMV-infected wild type *Arabidopsis* were more pronounced on the *ago2-1* mutants than wild type (Figure 3a). Associated with the enhanced symptoms, as in TCV-infected plants, the levels of the two forms of AGO2 increased (Figure 3b) and the level of viral RNA, assessed by quantitative RT-PCR, was higher (Figure 3c) than in wild type plants. However, unlike TCV, the increase in viral RNA persisted for at least 14dpi (Figure 3c).

*AGO2* mRNA is targeted by miRNA (miR403) in association with AGO1 [15], [16]. It is likely therefore that the induction of AGO2 in TCV- and CMV-infected plants (Figures 2 and 3) is because these viruses both produce suppressors of silencing that target AGO1. The CMV suppressor 2b targets and blocks the slicer activity of AGO1 [7] and the TCV suppressor P38 binds to and inactivates AGO1 [17]. The loss of AGO1 activity in the presence of these viruses would relieve the miR403-mediated suppression of *AGO2* mRNA.

**Figure 4:**
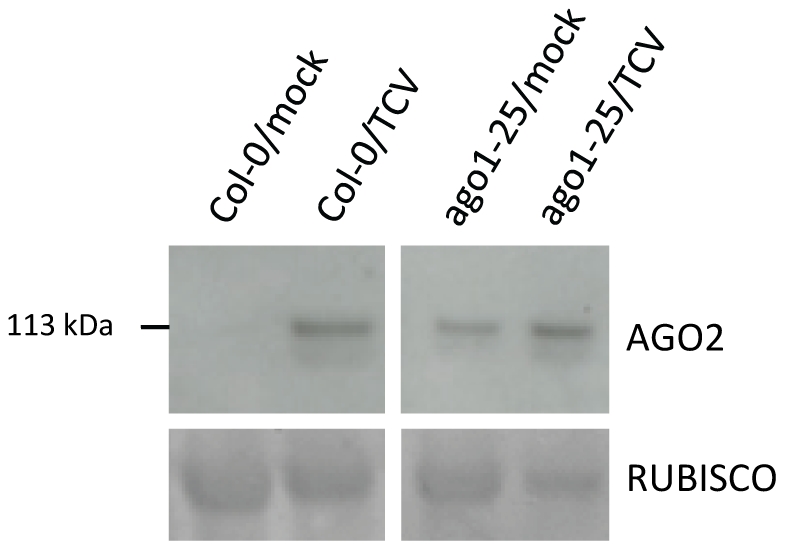
Induction of AGO2 by TCV is mimicked by loss of *AGO1* function. Western blot of AGO2 accumulation in mock- and TCV-infected WT (Col-0) and *ago1-25* mutants at 14 dpi. The two AGO2 panels are from different parts of the same western blot filter and they were both exposed together for the same length of time. Ponceau S-stained RUBISCO large subunit serves as the loading control.

To test this hypothesis we assayed AGO2 in extracts of non infected and TCV-infected *ago1-25* mutant and wild type Arabidopsis by western blotting. As predicted, in the non-infected plants, the level of AGO2 increased relative to wild type in the *ago1-25* mutant (Figure 4). The amount of AGO2 in the *ago1-25* mutant was similar to TCV-infected wild type plant and it did not increase further after TCV infection (Figure 4).

## Discussion

Although several AGO proteins have been associated with virus defense, the only definitive evidence for an antiviral role has previously been with AGO1 [12]. We now show that AGO2 also has an antiviral role against viruses that suppress AGO1. In effect AGO2 provides a secondary antiviral mechanism that is important when the primary AGO1-mediated layer is not active. Our analysis is therefore complementary to the previous elegant demonstration in which the first AGO1-dependent layer of defense was exposed through the use of a mutant TCV that did not produce the P38 suppressor of AGO1 [17].

Presumably the lack of an effect of AGO2 on susceptibility to TMV is because the suppressor of this virus [18] does not target AGO1 and AGO2 would not be induced. We predict that AGO2 would also not affect susceptibility to poleroviruses in which the suppressors of silencing target degradation of all AGO family members [19] or to other viruses with suppressors that target siRNAs and their precursors [20]. In contrast, we predict that AGO2 is likely to influence susceptibility to potexviruses because they encode a 25kDa protein that targets AGO1 [21].

How can the loss of AGO2 have a drastic effect on viral symptoms with only a small difference and transient effect on virus accumulation (Figures 1, 2, 3)? A similar result in which down-regulation of RDR6 in *Nicotiana benthamiana* resulted in enhanced symptoms of potato virus X but slight or no changes in overall virus accumulation was explained in terms of tissue specificity: symptoms are likely to be caused by virus in the growing point of the plant and RDR6 is required to prevent virus invasion of the meristem and growing points of the plant [22]. In this light it would be interesting to find out whether AGO2, like RDR6, is also involved in meristem exclusion of plant viruses. An alternative possibility is that AGO2 could have an effect in other cells, for example those in the vascular bundle, where suppression of virus accumulation might influence the symptoms.

The further understanding of how and when AGO proteins act in antiviral defense will be useful in the design of artificial resistance strategies. It will also be necessary to test our panel of *AGO* mutants against an extended set of mutant and wild type viruses to find out whether AGO proteins other than AGO1 and AGO2 have antiviral functions.

## Materials and Methods

### AGO mutants and growth conditions

The panel of TCV-inoculated mutants included the previously characterised *ago1-25* [12]; *ago 2-1* [16] (SALK_003380); *ago3-1* [16] (SM_3_31520); *ago4-3* [23] (WISC_338A06); *ago6-2* [24]; *ago 7-1* [25] (SALK_095997); *ago 9-1*, [26] (SALK_127358); and *pnh-2* (*ago10*) [26], [27]. PCR was used to verify these mutant genotypes before virus-inoculation.

In addition, previously uncharacterised alleles of *AGO5* and *AGO8* were used in this study. For *ago5-3* (SALK_063806) a T-DNA disrupts the splice donor of intron 16. Homozygous lines were confirmed using primers DBO373 5′-AGCATGGCTGTTCAAATAGAAGTC-3′and Lba1 5′-TGGTTCACGTAGTGGGCCATCG-3′ which detects the mutant *ago5-3* allele (approximately 570 bp) and DBO372 5′-ATCCACAACGTGGGCTAGTCC-3′and DBO373 which detects a wild-type allele (approximately 600bp). In *ago 8-2* (SALK_151983), a T-DNA insertion resides in exon 14. Homozygous lines were confirmed using primers DBO119 5′-CTTGGTGGATTGAATTCAGTTTTGG-3′ and Lba1 which detects the mutant *ago8-2* allele (approximately 350 bp) and DBO137 5′-CACTTACAATCTTTCCAG-3′and DBO119 which detects the wild-type allele (approximately 1000 bp). We assume that *ago5-3* and *ago8-2* are strong knock-out lines because the insertions disrupt their coding capacity. Further evidence that these are loss of function mutants is from the finding that *ago5-3* mutants have no detectable AGO5 protein and because *ago5-3* and *ago8-2* mutants have an effect on the expression of non coding RNAs that are the targets of endogenous siRNAs (E. Havecker, L. Wallbridge and DCB – in preparation for publication).

Appropriate wild type controls corresponding to the genetic background of each mutant were included. All plants were grown under short day conditions: 8hrs light at 200 micromol.m^−2^.s^−1^, 21°C, Conviron, Canada.

### CMV

*A. thaliana* plants were infected by rub inoculation using a 100 µg.ml^−1^ suspension of CMV particles [28]. Infection was confirmed by observation of symptoms. For analysis of viral titer in infected plants, protein was extracted and separated by SDS-PAGE, prior to transfer to nitrocellulose, as described previously [28], [29], [30]. Equal loading of gels was verified using Ponceau S staining of the large subunit of ribulose 1,5-bisphosphate carboxylase (RUBISCO), after which immunoblotting was conducted using rabbit antiserum against the CMV coat protein followed by an anti-rabbit IgG-horseradish peroxidase [28]. Antibody binding was observed by exposing the blot to a chemiluminescent peroxidase substrate followed with imaging on X-ray film [28].

### TCV

Infectious TCV RNA was *in vitro* transcribed from the pT7TCV clone from Anne Simon [31]. The RNA was rub-inoculated onto leaves of *N. benthamiana* and virus particles were purified from systemic, infected leaves at 28 dpi according to Díez *et al.* [32] except that 1% w/v ascorbic acid was added to the 0.2M sodium acetate solution, pH 5.0. Each *Arabidopsis* plant was rub-inoculated with carborundum using 5µl of a 1 µg. µl^−1^ suspension of TCV particles in 10 mM Tris-HCl pH 7.3. Buffer-only was similarly rub-inoculated as a control. Immunoblotting was conducted as for CMV, but using a 1∶10,000 dilution of primary antibody against TCV CP from Jack Morris.

### AGO2

For analysis of AGO2 protein abundance, protein was extracted as described previously [28]. Polyclonal AGO2 peptide antibodies were raised against the peptide sequence H2N-CGRKPQVPSDSASPSTST-CONH2 (Eurogentec; Seraing, Belgium). Immunoblotting was conducted with a 1∶4,000 dilution of the peptide-affinity purified anti-AGO2 antibody followed by a 1∶10,000 dilution of goat anti-rabbit IgG HRP conjugated secondary antibody (sc-2054, Santa Cruz Biotechnology). The polyclonal AGO2 antibody detects two bands of approximately 113 (predicted size of AGO2) and 108 kDa. These bands were not present in the *ago2-1* mutant indicating that the anti-AGO2 antibody is specific for AGO2 and that *ago2-1* is likely to be a protein null. Equal loading was verified and bound secondary antibody was detected as detailed for CMV coat protein, above.

#### Analysis of AGO2-bound sRNAs

AGO2-bound sRNAs in TCV-infected WT (Col-0) plants were immunoprecipitated as described previously [23]. These sRNAs were then cloned for Illumina sequencing as described previously [33], with the less than 200nt MirVana fraction used for Illumina library construction.

### Quantitative reverse transcription and polymerase chain reaction (Q-RT-PCR)

For quantification of CMV and TCV titer in WT and *ago2-1* mutants, whole aerial tissue was harvested from infected plants at 7 and 14 dpi. Total RNA for Q-RT-PCR analysis was extracted using TRIzol reagent (Invitrogen, Carlsbad, CA, USA) according to manufacturer's instructions. Total RNA was then further purified by lithium chloride precipitation and phenol-chloroform extraction [34] and subsequently treated with TURBO-DNase (Ambion) according to manufacturer's instructions. First strand synthesis was carried out with 0.5 µg total RNA using Superscript III (Invitrogen) with random hexamer primers according to manufacturer's instructions. Following the reaction, cDNA was diluted 1/5. Q-RT-PCR was performed using SYBR Green JumpStart Taq ReadyMix (Sigma) in 15 µl reactions according to manufacturer's instructions. Reactions were performed in triplicate. Primers were designed against the non-translated regions of the CMV and TCV genomes and a stable transcript of *AT3G50590* was used as a reference RNA. Data were analyzed using LinRegPCR to give Ct values and amplicon amplification efficiency [35], [36]. Relative virus accumulation was calculated using efficiency adjusted ΔΔCt methodology, incorporating the reference transcript to control for variation in loading [37], [38]. Virus accumulation was expressed relative to that in WT plants at 7 dpi.

TCV F 5′-aacggtggcagcactgtctagc-3′

TCV R 5′-ttggcttggaaggtcaccacagc-3

CMV F 5′-gtggaacgggttgtccatccagct-3′

CMV R 3′-cacccgtaccctgaaactagcacg-3′
